# Metastatic ovarian cancer spreading into mammary ducts mimicking an *in situ* component of primary breast cancer: a case report

**DOI:** 10.1186/s13256-020-02653-w

**Published:** 2021-02-17

**Authors:** Yurina Maeshima, Tomo Osako, Hidetomo Morizono, Mayu Yunokawa, Yumi Miyagi, Mari Kikuchi, Takayuki Ueno, Shinji Ohno, Futoshi Akiyama

**Affiliations:** 1 Division of Pathology, Cancer Institute of Japanese Foundation for Cancer Research, 3-8-31, Ariake, Koto-ku, Tokyo, 135-8550 Japan; 2 Department of Surgical Oncology, Breast Oncology Center, Cancer Institute Hospital of Japanese Foundation for Cancer Research, Tokyo, Japan; 3 Department of Pathology, Cancer Institute Hospital of Japanese Foundation for Cancer Research, Tokyo, Japan; 4 Department of Gynecologic Oncology, Cancer Institute Hospital of Japanese Foundation for Cancer Research, Tokyo, Japan; 5 Diagnostic Imaging Center, Cancer Institute Hospital of Japanese Foundation for Cancer Research, Tokyo, Japan

**Keywords:** Breast metastasis, Ovarian serous carcinoma, Invasive micropapillary carcinoma, *In situ* component, Case report

## Abstract

**Background:**

Accurate diagnosis of metastatic tumors in the breast is crucial because the therapeutic approach is essentially different from primary tumors. A key morphological feature of metastatic tumors is their lack of an *in situ* carcinoma component. Here, we present a unique case of metastatic ovarian carcinoma spreading into mammary ducts and mimicked an *in situ* component of primary carcinoma. To our knowledge, this is the second case (and the first adult case) confirming the *in situ*-mimicking growth pattern of a metastatic tumor using immunohistochemistry.

**Case presentation:**

A 69-year-old Japanese woman was found to have a breast mass with microcalcifications. She had a known history of ovarian mixed serous and endocervical-type mucinous (seromucinous) carcinoma. Needle biopsy specimen of the breast tumor revealed adenocarcinoma displaying an *in situ*-looking tubular architecture in addition to invasive micropapillary and papillary architectures with psammoma bodies. From these morphological features, metastatic serous carcinoma and invasive micropapillary carcinoma of breast origin were both suspected. In immunohistochemistry, the cancer cells were immunoreactive for WT1, PAX8, and CA125, and negative for GATA3, mammaglobin, and gross cystic disease fluid protein-15. Therefore, the breast tumor was diagnosed to be metastatic ovarian serous carcinoma. The *in situ*-looking architecture showed the same immunophenotype, but was surrounded by myoepithelium confirmed by immunohistochemistry (e.g. p63, cytokeratin 14, CD10). Thus, the histogenesis of the *in situ*-like tubular foci was could be explained by the spread of metastatic ovarian cancer cells into existing mammary ducts.

**Conclusion:**

Metastatic tumors may spread into mammary duct units and mimic an *in situ* carcinoma component of primary breast cancer. This *in situ*-mimicking growth pattern can be a potential pitfall in establishing a correct diagnosis of metastasis to the breast. A panel of breast-related and extramammary organ/tumor-specific immunohistochemical markers may be helpful in distinguishing metastatic tumors from primary tumors.

## Introduction

Breast cancer is the most common malignancy in women worldwide. However, metastases to the breast from extramammary solid tumors are rare and account for only 0.2–0.9% of all breast malignancies [1–3]. The most common primary tumors metastasizing to the breast vary depending on the specific patient population studied [4], but malignant melanoma, lung carcinoma, ovarian carcinoma, gastrointestinal carcinoma, and sarcoma are repeatedly reported [4–7].

Accurate diagnosis of metastatic tumors in the breast is crucial because their staging, treatment and prognosis are essentially different from primary breast tumors [6]. One of the key morphological features for the diagnosis of metastatic tumors is their lack of an *in situ* (intraductal and/or intralobular) carcinoma component [3, 5, 7]. The presence of an *in situ* component strongly supports the diagnosis of primary carcinoma. However, in this case report, we present a unique case of metastatic ovarian carcinoma spreading into mammary ducts, which mimicked an *in situ* component of primary breast carcinoma.

## Case presentation

### Clinical summary

**Fig. 1:**
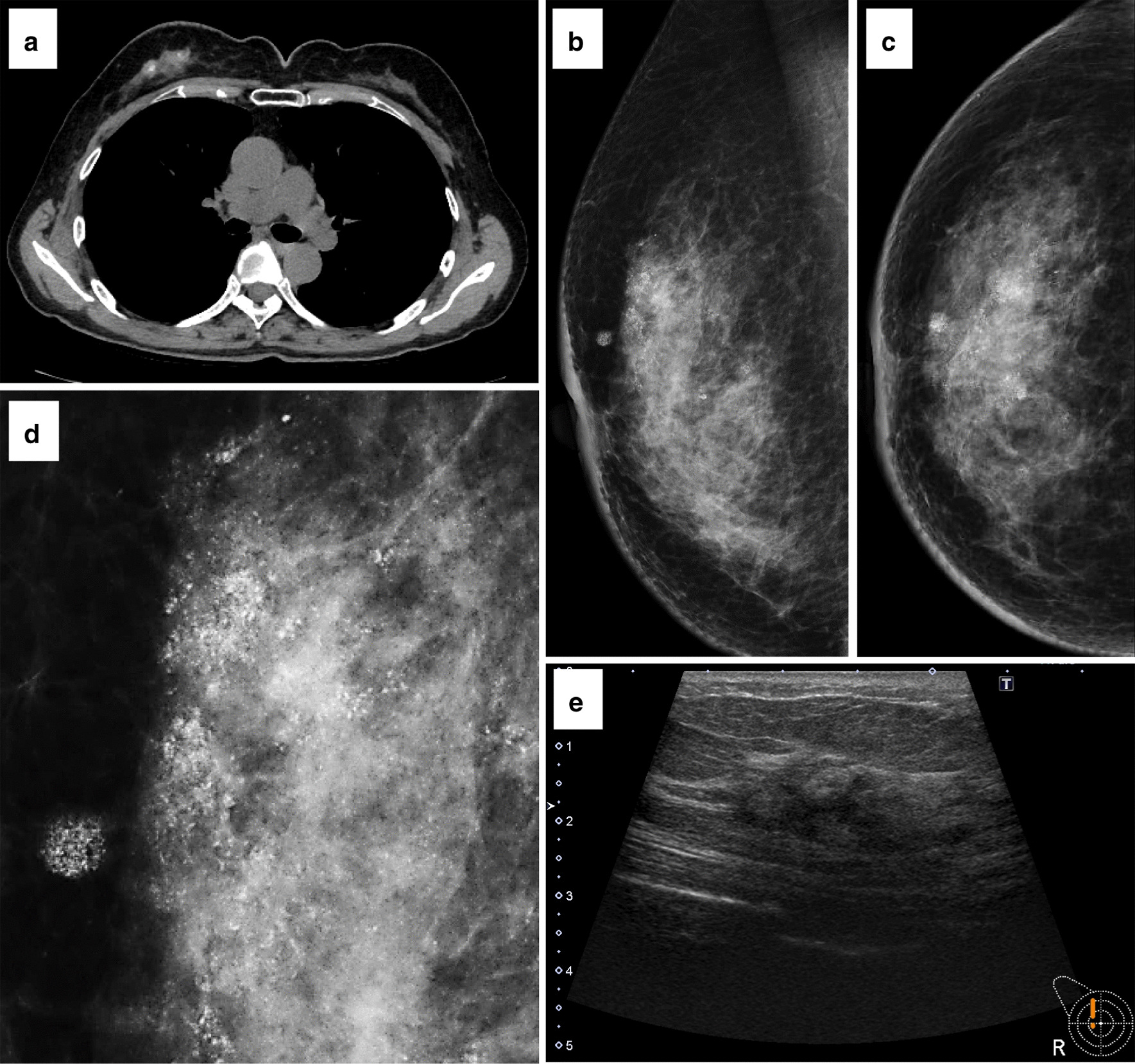
Radiological images of the metastatic ovarian serous carcinoma in the breast. Plain chest computed tomography (**a**); mediolateral-oblique and craniocaudal views of the mammography (**b**, **c**) and magnified image of the mediolateral-oblique view (**d**); and ultrasonography (**e**)

A 69-year-old Japanese woman was found to have a right breast mass with calcifications and pleural nodules on computed tomography for post-treatment surveillance of ovarian cancer (Fig. 1a). This patient had undergone debulking surgery and chemotherapy (carboplatin plus paclitaxel) for stage IIIc ovarian cancer 9 years before, and she had received additional chemotherapy (carboplatin plus paclitaxel) for bilateral axillary lymph node metastasis 4 years before. Then, she had been followed up every 3 months.

On physical examination, a 33 × 30-mm induration was palpable in the upper-outer quadrant of the patient’s right breast. Mammography showed segmental distribution of the amorphous microcalcifications associated with a focal asymmetric density in the upper-outer area of the breast (Fig. 1b–d). Ultrasonography showed a 33 × 33 × 16-mm irregular hypoechoic area with high echo spots and indistinct margins (Fig. 1e). From these images, ductal carcinoma *in situ* or invasive ductal carcinoma with a predominant intraductal component was primarily suspected, but metastatic ovarian cancer could not be excluded considering her clinical history. The patient underwent vacuum-assisted needle biopsy for the breast lesion. After the biopsy, she was treated with chemotherapy (carboplatin plus gemcitabine followed by carboplatin plus doxorubicin) for 2 years. At the time of this report (2 and half years after the biopsy), she receives best supportive care for metastatic ovarian cancer and myelodysplastic syndrome.

### Pathological findings of previous ovarian tumor

Macroscopic examination of the surgical specimens revealed a 65 × 55 × 45-mm, lobulated, whitish-yellow, solid mass in the right ovary; an 8-mm mass on the surface of the left ovary; and multiple disseminated tumors, up to 16 mm in size, in the greater omentum.

**Fig. 2:**
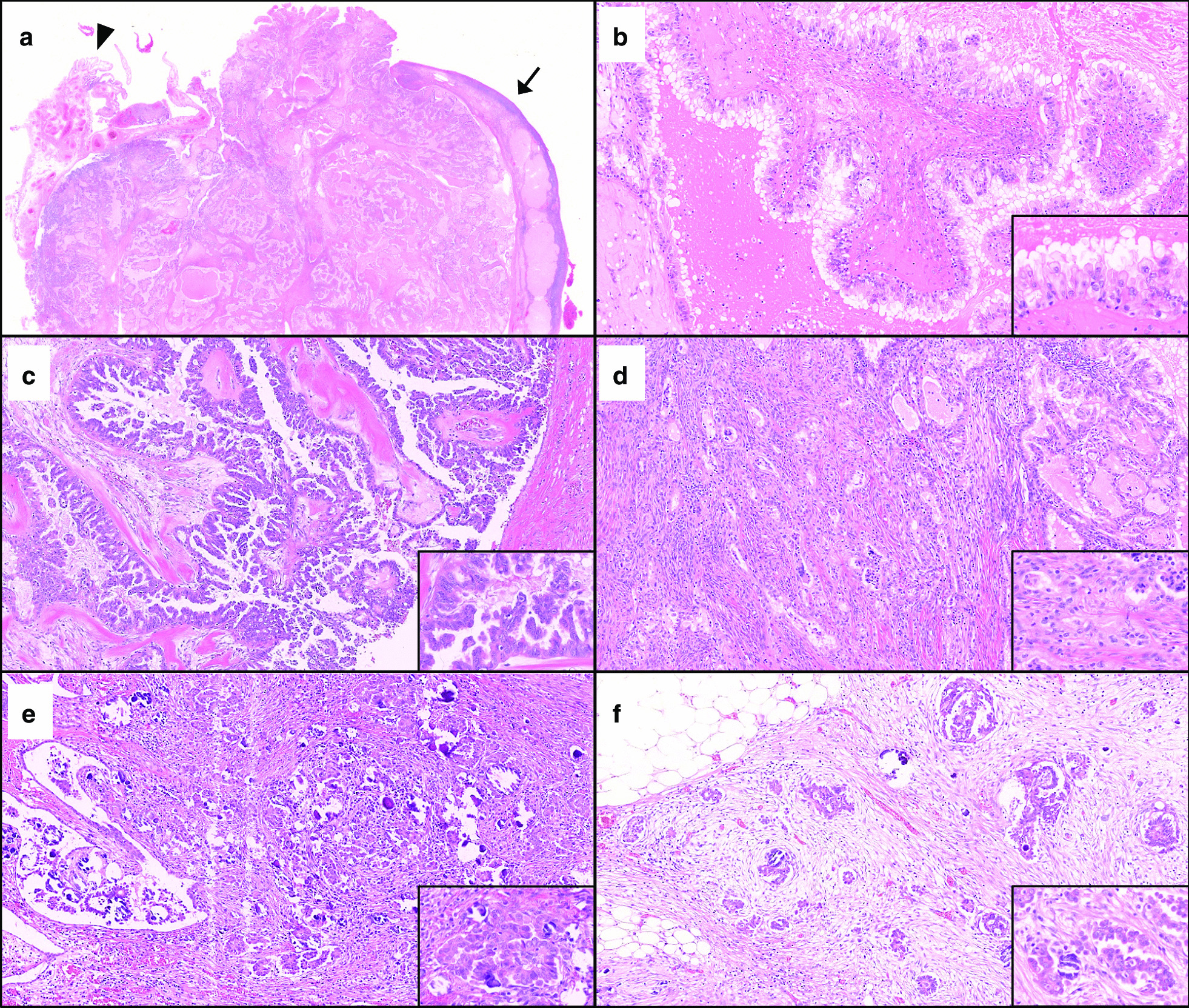
Pathological images of the primary ovarian tumor and omental dissemination. Hematoxylin-and-eosin-stainined images of the primary tumor: loupe view (**a** magnification ×5; arrow, ovary; arrow head, Fallopian tube), intermediate-magnification views of seromucinous borderline tumor (mixed endocervical-type mucinous tumor [**b**, ×100] and serous tumor [**c**, ×100]) and seromucinous carcinoma (mixed endocervical-type mucinous carcinoma [**d**, ×100] and low-grade serous carcinoma [**e**, ×100]. Hematoxylin-and-eosin-stainined images of omental dissemination of low-grade serous carcinoma (**f**, ×100).

Microscopically, the right ovarian tumor displayed a complex branching papillary architecture (Fig. 2a). The epithelium lining the papillae was stratified and was composed of endocervical-type mucinous epithelium (Fig. 2b) and serous epithelium (Fig. 2c). There were multiple invasive cancer foci in the stroma composed of endocervical-type mucinous epithelium displaying tubular architecture (Fig. 2d) and serous epithelium displaying micropapillary architecture with psammoma bodies (Fig. 2e). Thus, the tumor was diagnosed to be seromucinous carcinoma (mixed endocervical-type mucinous and low-grade serous carcinoma) associated with seromucinous borderline tumor. The immunohistochemical analysis supported the diagnosis (Table 1).

**Table 1.** Antibodies for immunostaining and results for the present case.

Antibody	Clone	Source	Dilution	Immunoreactivity		
				Ovarian carcinoma		Breast tumor
				Mucinous, endocervical type (seromucinous)	Serous, low grade	
WT1	WT49	Leica	1/30	(+)	(+++)	(+++)
PAX8	BC12	Nichirei	RTU	(+)	(+++)	(+++)
CA125	M11	Dako	1/50	(+++)	(+++)	(+++)
p53	DO-7	Dako	1/2000	wild type	wild type	wild type
ER	SP1	Ventana	RTU	(+++)	(+++)	(+++)
PgR	1E2	Ventana	RTU	(++)	(+)	(−)
GATA3	HG3-31	Santa Cruz	1/50	(−)	(−)	(−)
Mammaglobin	304-1A5	Dako	1/500	(−)	(−)	(−)
GCDFP15	D6	BioLegend	1/500	(−)	(−)	(−)
HER2	4B5	Ventana	RTU	(−)	(−)	(−)
Ki67	MIB1	Dako	1/50	15%	15%	15%

*ER* estrogen receptor, *PgR* progesterone receptor, *GCDFP15* gross cystic disease fluid protein-15, *HER2* human epidermal growth factor-2, *RTU* ready to use, *(+)* focal and weak staining, *(++)* intermediate staining, *(+++)* diffuse and strong staining

The left ovary was also diagnosed with seromucinous carcinoma with seromucinous borderline tumor. In the omentum, there were multiple disseminations of serous carcinoma accompanied with psammoma bodies and desmoplastic stroma (Fig. 2f).

### Pathological findings of breast tumor

**Figure 3.**
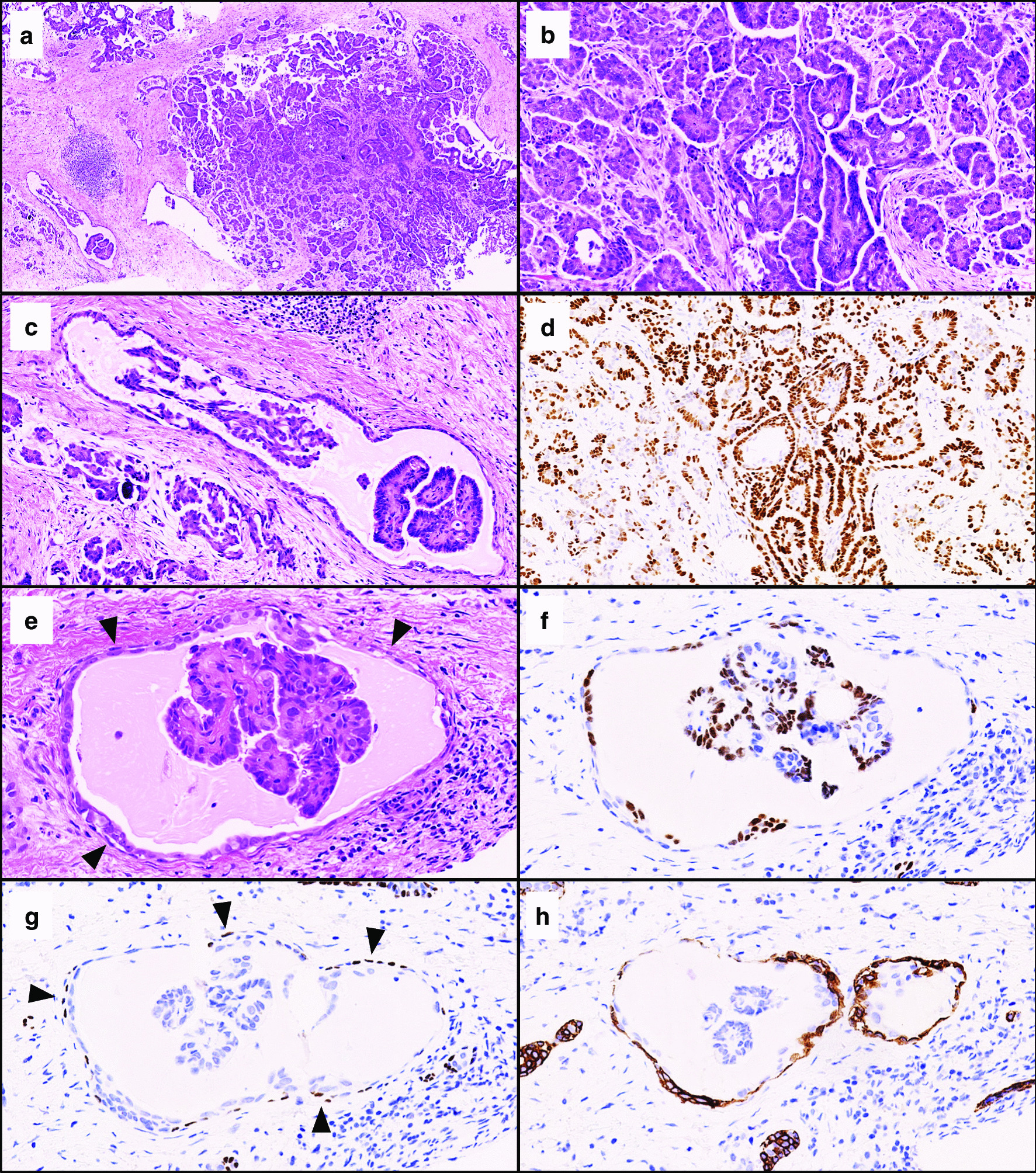
Pathological images of the metastatic ovarian serous carcinoma in the breast. Hematoxylin-and-eosin-stained images of the tumor: low-magnification view (**a**, magnification ×50), intermediate-magnification views of micropapillary/papillary architectures with psammoma bodies (**b**, ×200) and *in situ*-looking tubular architecture (**c**, ×200). Immunohistochemical image of GATA3 corresponding to the hematoxylin-and-eosin-stained image (**b**) (**d**, ×200). Immunohistochemical images of the *in situ*-looking structure corresponding to the hematoxylin-and-eosin-stained image (**e**; arrowhead, myoepithelium; magnification ×400): WT1 (**f**), p63 (g; arrowhead, myoepithelium) and cytoketatin 14 (h).

Microscopic examination of the needle biopsy specimen revealed invasive adenocarcinoma displaying micropapillary and papillary architectures in the breast stroma (Fig. 3a, b). The cancer cells displayed intermediate nuclear atypia and low mitotic activity. Psammoma bodies were frequently observed. In addition, a small number of cancer nests showed a tubular architecture surrounded by myoepithelium, which appeared to be an *in situ* carcinoma (Fig. 3a, 3c, 3e). On the hematoxylin-and-eosin-stained slides, metastatic serous carcinoma was primarily suspected due to the morphological similarities to the previous ovarian carcinoma, but invasive micropapillary carcinoma of the breast origin could not be ruled out considering the *in situ*-like foci.

In immunohistochemistry, the cancer cells were immunoreactive for WT1 (Fig. 3d), PAX8, and CA125, and negative for GATA3, mammaglobin, and gross cystic disease fluid protein-15 (GCDFP15) (Table 1). Therefore, the breast tumor was diagnosed to be metastatic ovarian serous carcinoma.

Regarding the *in situ*-like tubular foci, the cancer cells lining the tubule and papillae showed the aforementioned immunophenotype (e.g. positive WT1, Fig. 3f), but the tubular foci were surrounded by myoepithelium which was immunoreactive for myoepithelial markers [p63 (Fig. 3g), cytokeratin 14 (Fig. 3h), CD10 and calponin], and negative for endothelial markers (podoplanin and CD31). Thus, the histogenesis of the *in situ*-like tubular foci may be explained by the spread of metastatic ovarian cancer cells into existing mammary ducts.

## Discussion

We present a unique case of ovarian carcinoma metastasizing to the breast and spreading into mammary ducts, which mimicked an *in situ* component of primary breast carcinoma. One case report of metastatic pancreatic tumor in a child firstly confirmed this *in situ*-mimicking growth pattern by immunohistochemistry [8]. Two other reports briefly mentioned this growth pattern of metastatic tumors, but appropriate immunohistochemical stains to prove intraductal growth were not performed [2, 9]. Thus, to our knowledge, this is the second case (and the first adult case) confirming the growth pattern by immunohistochemistry. This *in situ*-mimicking growth pattern can be a potential pitfall for establishing a correct diagnosis of metastasis. The same growth pattern, aside from the metastatic tumor, was recently reported in a soft tissue tumor arising in the breast [10].

Histologically correct and type-specific diagnosis of tumors metastasizing to the breast is vital to ensure appropriate management. However, because of their rarity, it is sometimes difficult for pathologists to make the accurate diagnosis. The following four points can be given as diagnostic clues for metastatic tumors [2–5, 7, 11, 12]: (1) clinical history of extramammary malignancy, (2) unusual morphology for primary breast cancer, (3) absence of an *in situ* component, and (4) lack of breast-related immunophenotype.

Clinical history of extramammary cancer is essential in making a diagnosis of metastasis to the breast [3, 5, 11]. Almost all breast cancer cases that pathologists diagnose in daily practice are primary cancers. Thus, suspicion of metastatic tumors may sometimes be raised only after clinical history is provided. Comparison of mammary and extramammary tumors is important in this situation.

The diagnosis of metastatic tumors is easier when the tumor has an unusual appearance for a breast primary lesion or a typical morphology of its primary site of origin [12]. Two-thirds of metastases to the breast have distinctive histological features, raising the possibility of the diagnosis [3]. In remaining cases, however, the histological appearance is similar to a primary mammary tumor, and the clinical history and other information are important to establish the correct diagnosis for these cases.

The presence of an *in situ* carcinoma component is pathognomonic of primary breast carcinoma. On the contrary, the absence of an *in situ* component supports the diagnosis of a metastatic tumor to the breast [3, 5, 7]. However, *in situ*-like atypical ductal proliferations are reported in metastatic tumors in the breast, and pathologists should be careful not to regard this *in situ*-like structure as true *in situ* carcinoma and exclude the possibility of metastatic tumor. The *in situ*-mimicking lesions in metastatic tumors can be classified into three types based on histogenesis: (a) lymphovascular emboli from metastatic tumors [2, 11], (b) metastatic tumors spreading into existing mammary duct units [2, 8, 9], and (c) true *in situ* carcinoma or atypical ductal/lobular hyperplasia of breast origin coexisting with metastatic tumors [9, 12]. The immunohistochemical panel mentioned below can be useful for the differential diagnosis (Table 2).

**Table 2.** Immunohistochemical markers for the differential diagnosis of *in situ*-mimicking architectures in metastasis to the breast

	Extramammary organ/tumor-specific	Breast-related	Myoepithelial	Lymphatic endothelial
Lymphovascular tumor emboli	(+)	(−)	(−)	(+)
Intraductal spread by metastatic tumors^a^	(+)	(−)	(+)	(−)
Coexistence of true *in situ* carcinoma	(−)	(+)	(+)	(−)

^a^The present case

Immunohistochemistry plays a major role in the accurate diagnosis of metastatic tumor in the breast. A panel of breast-related markers (e.g., GATA3, mammaglobin, GCDFP15, and SOX10) is helpful to rule out a metastasis [5]. In addition, a panel of extramammary organ/tumor-specific markers can be used to delineate the likely primary site of metastasis [5]. In addition, myoepithelial markers (e.g. p63, cytokeratin 14, and calponin) and endothelial markers (e.g., podoplanin and CD31) may be useful for the differential diagnosis of *in situ*-like architecture in the metastatic tumors [4, 5, 11].

Serous carcinoma is the most common type of ovarian tumor metastasizing to the breast [13]. Metastatic serous carcinoma can resemble invasive micropapillary carcinoma of the breast, and psammomatous calcifications can be seen in both [3, 14, 15]. One study reported that approximately 24% of metastatic serous carcinomas in the breast were initially interpreted as primary carcinomas [14]. In fact, invasive micropapillary carcinoma was originally designated “pseudopapillary (serous-like) carcinoma” because of its resemblance to serous carcinoma of Müllerian origin [15]. A key morphological finding for the differential diagnosis may be the presence or absence of a fibrovascular core in the papillary/micropapillary structure. Invasive micropapillary carcinoma mainly shows micropapillary (pseudopapillary) architecture without fibrovascular cores [15], while serous carcinoma often displays (macro-)papillary structure with fibrovascular cores in addition to their micropapillary architecture [16]. A panel of breast-related and Müllerian duct/serous tumor-specific immunohistochemical markers (e.g. WT1 and PAX8) can be helpful in differentiating tumors of ovarian from breast origin. However, estrogen and progesterone receptors are not helpful because both tumors can be positive for these [13].

In the present case, immunohistochemistry played a critical role in establishing an accurate diagnosis of the metastatic tumor and in elucidating the histogenesis of its *in situ*-mimicking architecture. Although the breast tumor was histologically similar to the previous ovarian carcinoma, invasive micropapillary carcinoma of breast origin could not be ruled out because the *in situ*-like component surrounded by myoepithelium was present. Based on the positive Müllerian/serous markers and negative breast-related markers, the breast tumor and the *in situ*-like component was diagnosed as metastatic serous carcinoma. The histogenesis of the *in situ*-like architecture could be due to spread of the metastatic tumor into existing mammary ducts, since myoepithelium around the *in situ*-like component was confirmed by myoepithelial immunomarkers.

## Conclusions

We present a unique case of metastatic ovarian carcinoma spreading into mammary ducts, which mimicked an *in situ* component of primary breast carcinoma. This *in situ*-mimicking growth pattern of metastatic tumors can be a potential pitfall in establishing a correct diagnosis of metastasis to the breast. A panel of breast-related and extramammary organ/tumor-specific immunohistochemical markers may be helpful in distinguishing metastatic tumors from primary tumors.

## Data Availability

Not applicable.

## Abbreviation

GCDFP15: Gross cystic disease fluid protein-15
